# Meteorological Table

**Published:** 1846-03

**Authors:** B. F. Meek, A. J. Munsell


					﻿meteorological Mean, for the half year ending December, 1815.
BY B. F. MEEK AND A. J. MUNSELL.
Station, Frankfort, Ky.—Lat. 38° 14' N. Long. 7° 50' West from Washington.
barometer.	Thermometer^ Uranoscop a. Dew-point' Pluviometer.-
"5	- U i §	« ?-'! = 1	.	® I f i -s 5
MONTHS.	-g	g	g	g	g	o	q 8 s	g	&	1	xlH	’	*	'£
a	P	a	*	gs	=	=*ac^	=	c5S	'u	h * c c	.£	m	cs
Vj	fig__ W a rp *	%	£ Q o	>	fl S S pi	g	S*	Q»
July,....... x9.6u 2. .0* x9.6i 29.b5| 29.25 00.60 75 82 78 90 6‘ ~2 T ~2	29~ 6< '.3 7- j!8|26	5 j .5j l.,u
August,..... 29.71	j 29-69	29.70	30.05	29.45	00.60	73 81 78 88 58 ;■	0	3	I	28	67 73 80 55 25	10	|	125 I 7.45
September...	29.77	j 29.73	29.73	30.05	29 35	00.70	62 78 74 86 46 4(1	1	|	28	58 67 76 42 34	6	I	3.25	7.25
October,.... 29 99	| 29.93	29.85	30.48	29.42	01.06	47 66 60 76 34 4	4	2	25	48 55 68 35 33	|	8	|	1.25	5.80
November,...	29.79	29.74	29.56	30.35	20.07	01.28	37 53 49 68 12 56	1	3	26	36 45 55 30 25	j	~|	1.50	5.65
December....	2 .96	29.90	29.90	30.47	29.10	01.37	20 32 28 50 -2 j5:	5	10	|	16	35 39 46 34 12	|	3	|	.25	.55
S. A. Menn,... 29.81 29,77 29.73 \ 30,21' 29,27 00.93 52(65 61 76 36;4l( 11 20 j~53 52,59 61 1126^ 39 1.67 28.40 ^
The days marked “clear” and “cloudy” were entirely so during the twenty-four
hours. The Barometer and Thermometer were suspended in the shade with a
Southern exposure. The Dew-point is the highest temperature at which the vapor
in the open air will condense upon a bright metallic or thin glass tumbler of water,
cooled down by ice or pulverized hydrochlorate of ammonia and nitrate of potash in
equal quantities. It was carefully ascertained.
				

